# Primary Hypothyroidism and Alzheimer’s Disease: A Tale of Two

**DOI:** 10.1007/s10571-023-01392-y

**Published:** 2023-08-04

**Authors:** Faisal Holil AlAnazi, Hayder M. Al-kuraishy, Athanasios Alexiou, Marios Papadakis, Mohamed H. Mazhar Ashour, Saud A. Alnaaim, Omnya Elhussieny, Hebatallah M. Saad, Gaber El-Saber Batiha

**Affiliations:** 1 Department of Medicine, College of Medicine, Majmaah University, Majmaah, Saudi Arabia; 2 Department of Pharmacology, Toxicology and Medicine, Medical Faculty, College of Medicine, Al-Mustansiriyah University, P.O. Box 14132, Baghdad, Iraq; 3 Department of Science and Engineering, Novel Global Community Educational Foundation, Hebersham, NSW 2770 Australia; 4 AFNP Med, 1030 Vienna, Austria; 5 Department of Surgery II, University Hospital Witten-Herdecke, University of Witten-Herdecke, Heusnerstrasse 40, 42283 Wuppertal, Germany; 6 General & Pediatric Surgery, Faculty of Medicine, Tanta University, Tanta, Egypt; 7 Clinical Neurosciences Department, College of Medicine, King Faisal University, Hofuf, Saudi Arabia; 8 Department of Histology and Cytology, Faculty of Veterinary Medicine, Matrouh University, Marsa Matruh, 51744 Egypt; 9 Department of Pathology, Faculty of Veterinary Medicine, Matrouh University, Marsa Matruh, 51744 Egypt; 10 Department of Pharmacology and Therapeutics, Faculty of Veterinary Medicine, Damanhour University, Damanhour, 22511 Egypt

**Keywords:** Hypothyroidism, Alzheimer’s disease, Thyroid-stimulating hormone, Oxidative stress, Mitochondrial dysfunction

## Abstract

Hypothyroidism (HPT) HPT could be a risk factor for the development and progression of Alzheimer’s disease (AD). In addition, progressive neurodegeneration in AD may affect the metabolism of thyroid hormones (THs) in the brain causing local brain HPT. Hence, the present review aimed to clarify the potential association between HPT and AD. HPT promotes the progression of AD by inducing the production of amyloid beta (A*β*) and tau protein phosphorylation with the development of synaptic plasticity and memory dysfunction. Besides, the metabolism of THs is dysregulated in AD due to the accumulation of A*β* and tau protein phosphorylation leading to local brain HPT. Additionally, HPT can affect AD neuropathology through various mechanistic pathways including dysregulation of transthyretin, oxidative stress, ER stress, autophagy dysfunction mitochondrial dysfunction, and inhibition of brain-derived neurotrophic factor. Taken together there is a potential link between HPT and AD, as HPT adversely impacts AD neuropathology and the reverse is also true.

**Graphical Abstract:**

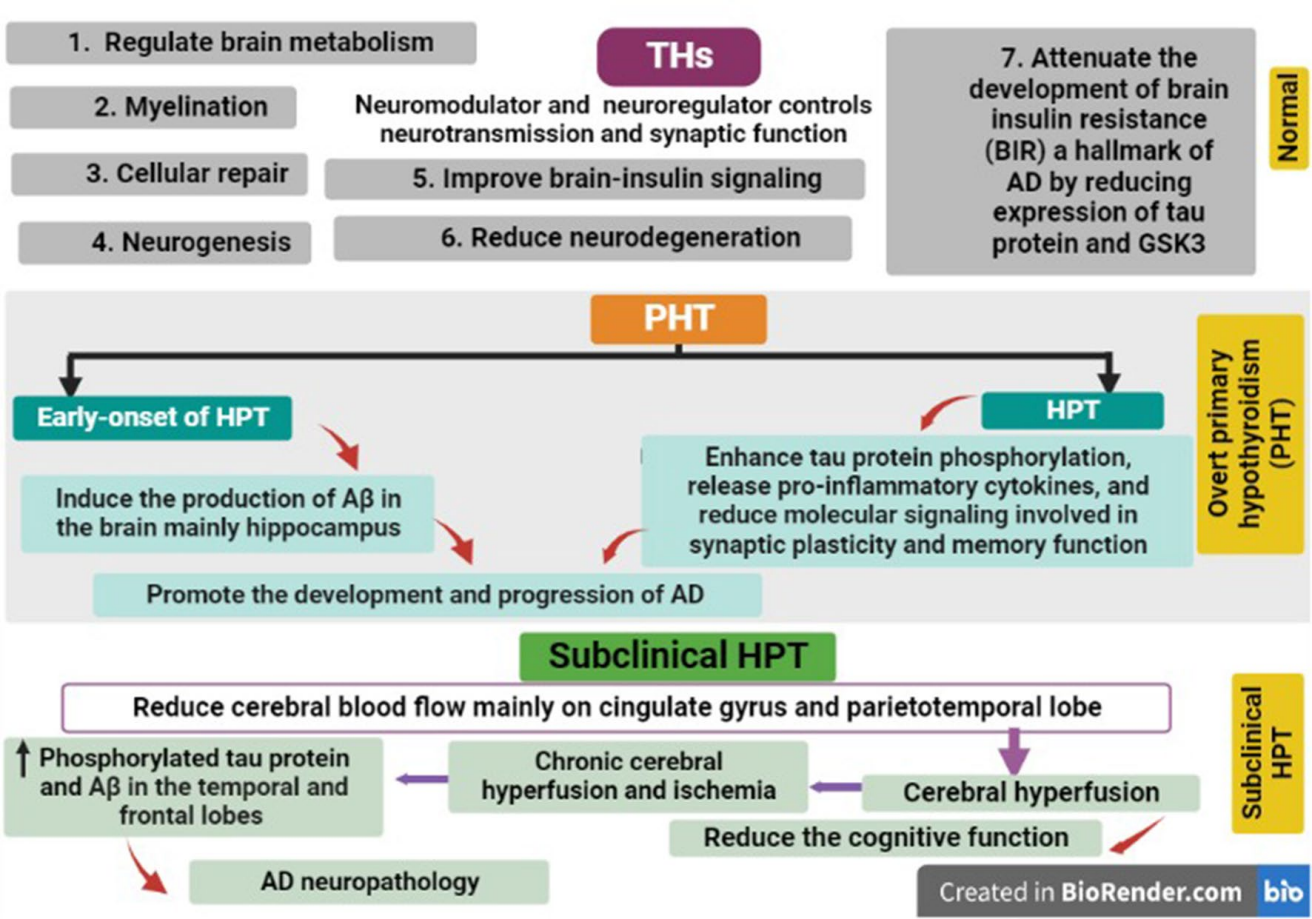

## Introduction

**Fig. 1:**
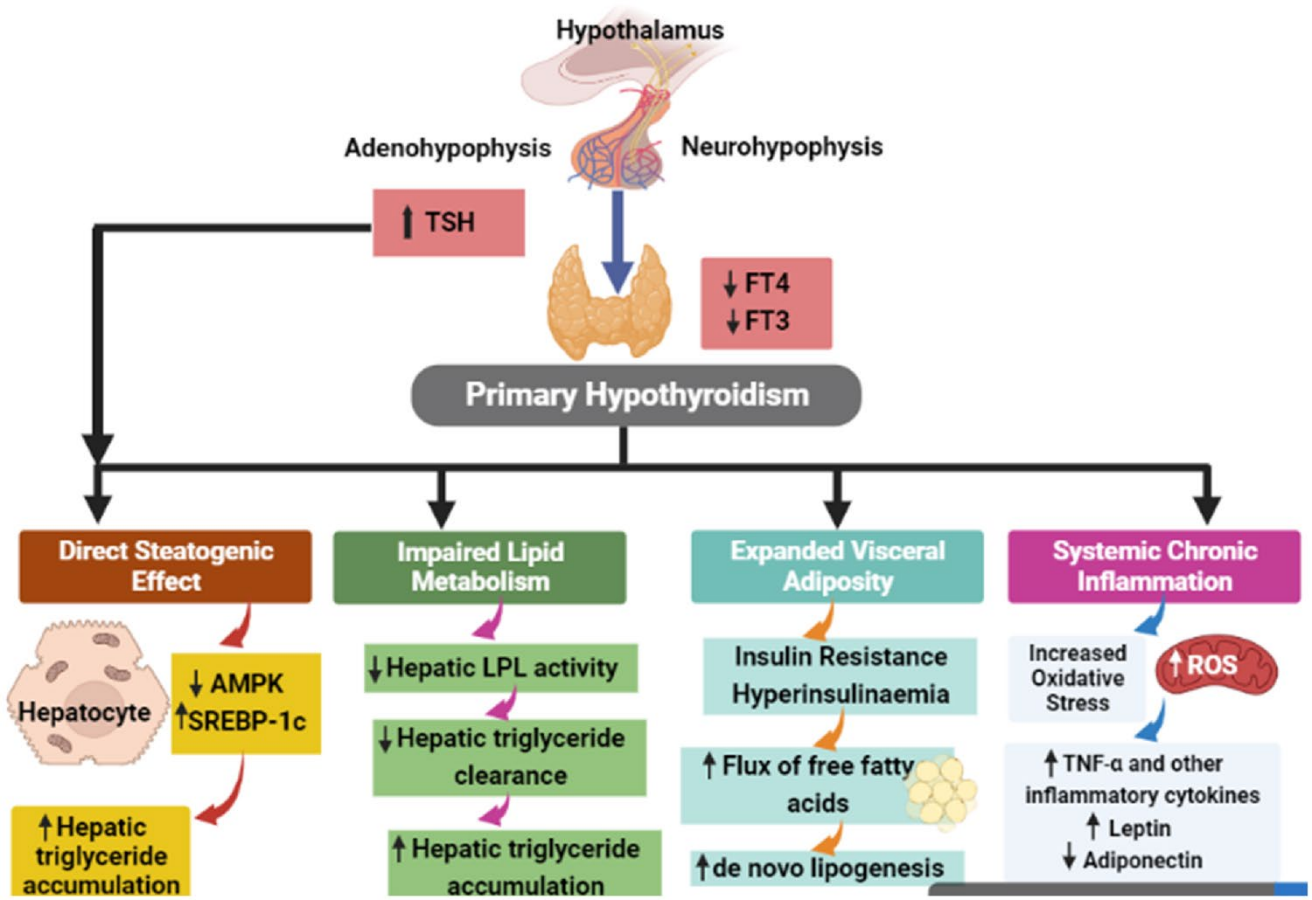
Systemic effects of hypothyroidism (HPT): In primary HPT, both free T3 and T4 are reduced leading to impairment of lipid metabolism, expansion of visceral fat and the development of systemic inflammatory disorders. These metabolic changes increase hepatic triglyceride accumulation, augmentation of lipogenesis and leptin with a reduction of anti-inflammatory adiponectin. Besides, increasing the release of thyroid-stimulating hormone from adenohypophysis due to PHT induces the accumulation of hepatic triglyceride

Hypothyroidism (HPT) is an endocrine disease due to insufficient function of the thyroid gland to produce adequate thyroid hormones (THs) to preserve the body’s metabolism (Al-Naimi et al. 2018). HPT could be symptomatic in the clinical type or asymptomatic in the subclinical type. Clinical or overt HPT is characterized by specific clinical features including cold intolerance, poor appetite, weight gain, bradycardia, constipation, and depression (Abdul-Hadi et al. 2020; Abdulhadi et al. 2021). Though, subclinical HPT which is characterized by normal TH levels and high thyroid-stimulating hormone (TSH) is chiefly asymptomatic (Al-Kuraishy et al. 2021; Al-Naimi et al. 2018). Subclinical HPT is regarded as a compensated state in which high TSH sustains normal thyroid function (Al-Kuraishy et al. 2021; Al-Naimi et al. 2018). THs including thyroxine (T4) and triiodothyronine (T3) are essential for brain growth and development therefore the reduction of these hormones during fetal life leads to congenital hypothyroidism (CH) (Rastogi and LaFranchi 2010). Typically, CH is diagnosed at birth with a high TSH level, if untreated following birth leads to growth failure and intellectual disability (Wassner 2018). The main causes of HPT are iodine deficiency which is required for the synthesis of THs (Wartofsky and Klubo-Gwiezdzinska 2019; Zimmermann and Boelaert 2015), autoimmune diseases by an autoantibody against TSH receptors (Ragusa et al. 2019), postpartum thyroiditis (Nguyen and Mestman 2019) and iatrogenic due to surgery, radiotherapy extends the use of certain drugs like interferon and amiodarone which interfere with iodine and synthesis of THs (Nguyen and Mestman 2019). HPT leads to systemic adverse effects including impairment of lipid homeostasis, steatogenic effect, expansion of visceral fat, and induction of chronic inflammation (Mantovani et al. 2018). These changes trigger the development of insulin resistance (IR), inhibition of hepatic lipoprotein lipase (LPL) activity, and augmentation of oxidative stress and inflammatory disorders (McAninch et al. 2018) (Fig. 1).

**Fig. 2:**
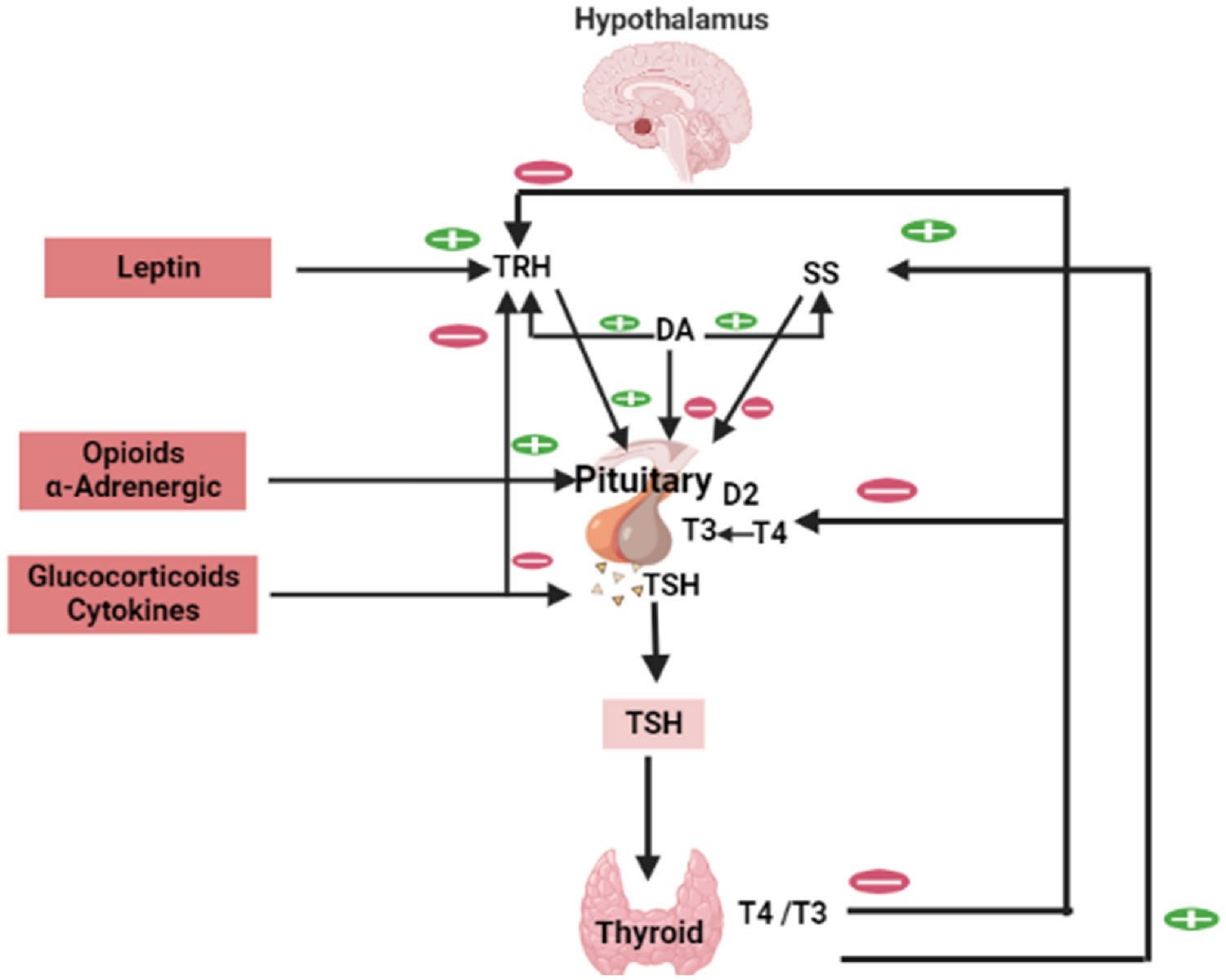
Effect of thyroid-stimulating hormone (TSH): Thyrotropin-releasing hormone (TRH) from the hypothalamus activates the release of TSH from the anterior pituitary which activate the release of thyroid hormones (THs) in a positive feedback loop. Increasing levels of THs inhibit the release of both TRH and TSH in a negative feedback loop. Leptin activates the release of TRH from the hypothalamus; somatostatin (SS) and dopamine (DA) inhibit TSH release from the pituitary. Glucocorticoids and pro-inflammatory cytokines inhibit TSH release

TSH is a glycoprotein hormone released from the anterior pituitary under the influence of thyrotropin-releasing hormone (TRH) from the hypothalamus (Fan et al. 2022). TSH encourages the thyroid gland to synthesize and release of THs. THs inhibits the release of TSH and TRH from the pituitary and hypothalamus correspondingly in a negative feedback loop (Fan et al. 2022). TSH is higher during the growth period and activated by stress (Fan et al. 2022). Furthermore, leptin activates the release of TRH from the hypothalamus; though somatostatin (SS) and dopamine (DA) inhibit TSH release from the pituitary (Gordon et al. 2010). In addition, glucocorticoids and pro-inflammatory cytokines inhibit TSH release (Gordon et al. 2010) (Fig. 2).

TSH acts on specific receptors called TSHRs which are G-protein coupled seven-transmembrane receptors (Williams 2011). TSHRs are primarily expressed on the thyroid epithelial cells, anterior pituitary, and hypothalamus intricate in the regulation of THs release and TSH response (Williams 2011). Extra-thyroid expression of TSHRs includes skin, ovary, immune system, kidney, peripheral blood cells, bone marrow, adipose tissue, bone and endothelial cells. Systemic expression of TSHRs proposes a role of TSH in diverse cardiometabolic processes and inflammatory reactions irrespective of the thyroid gland (Sun et al. 2015).

It has been shown that THs are necessary for brain development and the improvement of cognitive function. THs and TSH regulate brain metabolic function, neurotransmitter release and synaptic activity (Guedj et al. 2005; Salehipour et al. 2023). THs improve neurogenesis, and myelination, and inhibition of T3 reduces brain neurogenesis and development. Of interest, brain T3 is formed locally from T4 by the action of type 2 deiodinase which expressed in glial cells (Guedj et al. 2005; Salehipour et al. 2023). However, type 3 deiodinase which expressed by neurons degrade THs to inactive metabolites. Therefore, brain THs are regulated and conserved to control expression of transcription factors and genes related to regulate mitochondrial function and myelin formation (Guedj et al. 2005; Salehipour et al. 2023). Therefore, HPT may adversely affect the brain metabolic function and induce neuronal hypometabolism which is implicated in the development of neurodegenerative diseases such as Alzheimer’s disease (AD) (Guedj et al. 2005; Salehipour et al. 2023). Besides, AD can affect the development of HPT (Yong-Hong et al. 2013). Most clinical studies evaluate the effect of HPT on AD risk; however, the novelty of the present review was to find the association between HPT and the molecular mechanisms involved in the development of AD.

## Method and Search Strategy

According to the PRISMA guideline, we search different search engines including PubMed, Scopus, Embas and Google scholar databases by using keywords (Hypothyrodism AND Alzheimer disease), (Hypothyrodism AND Cognitive dysfunction), (Hypothyrodism AND Amyloid protein). Following each search in the database, we include and exclude different studies according to their eligibility in this review. All of retrieved articles of English language were included, though a case-report studies were excluded.

## Overview of Alzheimer’s Disease

AD is an advanced neurodegenerative disease characterized by cognitive dysfunction and memory loss. AD is the most common type of dementia; represents two-thirds of all dementia types in patients aged more than 65 years (Alsubaie et al. 2022). AD may be inherited as autosomal dominant due to mutation in the amyloid beta precursor protein (APP) gene which increases the production of amyloid beta (A*β*), and the presenilin 1 (PS1) gene which promotes the aggregation of A*β* (Al-Kuraishy et al. 2023b). Two types of AD are recognized, early-onset AD started before the age of 65 years and accounts for 10% of all AD cases, and late-onset AD started after 65 years and represents 90% of all AD cases (Al-Kuraishy et al. 2023a). Mutations of APP and PS1 genes trigger the progress and of early-onset AD (Novikova et al. 2021). Notably, apolipoprotein E4 (ApoE4) which controls lipid metabolism has a higher affinity to A*β* and is concerned with AD neuropathology (Najm et al. 2019). ApoE4 mutation prompts the development of sporadic and familial AD. ApoE4 heterozygote and homozygote increase AD risk by 50 and 90%, respectively. Although, the occurrence of ApoE4 does not constantly persuade the progress of AD (Parhizkar and Holtzman 2022).

AD prodromal symptoms are loss of short-term memory with conservation of long-term memory. Progressive deterioration of executive function and motivation disorders are also developed (Wang et al. 2022). A range of neuropsychiatric disorders, including agitation, olfactory dysfunction, dyspraxia, sleep disorders, and motor dysfunctions are developed during AD neuropathology (Yu et al. 2021; Zhang et al. 2022). Old age, smoking, depression, family history, head injury, and cardiometabolic disorders are the main risk factors for the development of AD (Silva et al. 2019). Nonetheless, estrogen, education, exercise, the use of anti-inflammatory drugs, and regular diets are considered the main protective factors against AD.

AD neuropathology is characterized by the neuronal deposition of neurofibrillary tangles and neuritic plaques (Camacho et al. 2022). AD plaques consist of intracellular neurofibrillary tangles and extracellular A*β* peptides (Rahman and Lendel 2021). Normally, A*β* peptide which is generated from APP by the action of α and *β* secretases is not toxic to the neurons (Leong et al. 2020). Nonetheless, sequential cleavage of A*β* peptide by γ secretase induces the generation of neurotoxic A*β*_1-42_ which is prone to aggregation and forms A*β* plaques and neurofibrillary peptide (Mansor et al. 2021). Furthermore, the tau protein which stabilizes neuronal microtubules and transport is also affected in AD (Guo et al. 2020). Extracellular deposition of A*β* plaques prompts phosphorylation of tau protein leading to tau aggregates that form intracellular neurofibrillary tangles which are more associated with AD neuropathology than the A*β* peptide (Zhang et al. 2021). Moreover, AD neuropathology is also associated with the formation of granule-vacuolar degeneration of the hippocampus by A*β*-induced angiopathy (Cajamarca et al. 2020). These neuropathological changes provoke advanced neurodegeneration and loss of cholinergic neurotransmission which are linked with cognitive dysfunction.

## Hypothyroidism and AD

### Effects of Hypothyroidism on AD

THs play important roles in the regulation of brain metabolism, myelination, cellular repair, and neurogenesis. TH receptors are highly expressed in the brain mainly in the cholinergic neurons of the hippocampus and basal forebrain. THs are regarded as neuromodulators and neuro-regulators control neurotransmission and synaptic function. Therefore, dysregulation of THs may induce remarkable brain dysfunction including memory and cognitive dysfunctions (Bauer et al. 2008; Khaleghzadeh-Ahangar et al. 2022). THs affect the pathogenesis of AD and the development of cognitive dysfunction (Khaleghzadeh-Ahangar et al. 2022). However, peripheral thyroid status does not exactly correspond with the central effects of THs as neuropsychiatric disorders may develop with mild alterations of THs. Furthermore, overt and subclinical HPT is regarded as a potential risk factor for the development of AD (Tan et al. 2008).

Preclinical studies demonstrated a potential effect of THs on AD neuropathology. THs inhibit gene expression of APP thereby reducing A*β* formation through reducing histone H3 acetylation and methylation (Belakavadi et al. 2011). It has been shown that HPT is regarded as a potential risk factor for the development of AD (Chaalal et al. 2014). Early-onset primary HPT promotes the development and progression of AD by inducing the production of A*β* in the brain mainly in the hippocampus (Chaalal et al. 2014). HPT enhances tau protein phosphorylation, the release of pro-inflammatory cytokines and the reduction of molecular signaling involved in synaptic plasticity and memory function (Chaalal et al. 2014). Therefore, THs were suggested to be an effective therapeutic strategy against AD neuropathology through the restoration of memory and cognitive functions (Bavarsad et al. 2019).

Remarkably, even subclinical HPT may affect the pathogenesis of AD through the modulation of cerebral blood flow (Haji et al. 2015). A cohort study on 11 AD patients with subclinical HPT and 141 AD patients without subclinical HPT revealed that cerebral blood flow mainly in the cingulate gyrus and parieto-temporal lobe was reduced in subclinical HPT compared to the controls (Haji et al. 2015). Therefore, subclinical HPT may reduce cognitive function through the induction of cerebral hypoperfusion. Park et al. (2019) observed that chronic cerebral hypoperfusion and ischemia promote AD neuropathology by increasing the generation of phosphorylated tau protein and A*β* in the temporal and frontal lobes, respectively. Besides, chronic cerebral hypoperfusion reduces brain glucose metabolism in mice (Park et al. 2019). Therefore, HPT contributes to the pathogenesis of AD by induction of cerebral hypoperfusion.

Reduction of THs is linked with the development of cognitive dysfunction as in euthyroid sick syndrome (Mafrica and Fodale 2008). A previous cohort study on 194 patients with AD and subclinical HPT compared to 122 controls showed that TSH level was increased in AD patients (Ganguli et al. 1996). Therefore, subclinical HPT is regarded as a risk factor for the development and progression of AD. TSHRs are highly expressed in the brain mainly in the limbic system and are associated with the development of different brain disorders such as bipolar disorders and depression (Naicker et al. 2019; Naicker and Naidoo 2022). Different clinical studies indicated that a dysregulated TSH serum level was correlated with AD neuropathology by increasing A*β* and phosphorylation of tau protein (Choi et al. 2020; Nomoto et al. 2019).

Different studies showed that a reduction of T3 is associated with atrophy of the brain cortex and hippocampus with the development of synaptic dysfunction and cognitive dysfunction (Montero-Pedrazuela et al. 2006). Finding from a preclinical study observed that administration of T3 retard A*β* pathology and associated neuroinflammation (Chaalal et al. 2019). Moreover, THs improve brain insulin signaling in the hippocampus and reduce neurodegeneration (Prieto-Almeida et al. 2018). THs attenuate the development of brain insulin resistance (BIR) a hallmark of AD by reducing the expression of tau protein and glycogen synthase kinase 3 (GSK3) (Prieto-Almeida et al. 2018). Likewise, administration of T4 attenuates neuroinflammation, A*β* pathology and memory impairment in diabetic rats (Chaalal et al. 2014). In the clinical setting, the association between HPT and AD risk remains controversial regarding age and sex (Mathew et al. 2020; Salehipour et al. 2023).

These findings indicated that HPT promotes the development and progression of AD neuropathology by increasing the expression of tau protein and aggregation of A*β*.

### Effects of AD on Thyroid Function

It has been shown that AD neuropathology affects thyroid function. The underlying association between AD and HPT is complex, as HPT is the cause of AD, or HPT as a secondary outcome for AD remains not well-identified. In advanced AD with the involvement of the hypothalamus and anterior pituitary, the hypothalamic-pituitary-thyroid axis is deregulated leading to HPT (Yong-Hong et al. 2013). However, Du and Pang (2015) showed that the accumulation of A*β* in early AD contributes to the dysregulation of the hypothalamic-pituitary axis and the development of neuropsychiatric disorders in the prodromal phase. A population-based study comprised 1077 elderly individuals started in 1995 and followed till 2005 showed that 46 of them developed AD which was not related to the TSH level (de Jong et al. 2006). This outcome proposed that high TSH in HPT is not implicated in the pathogenesis of AD. A systematic review and meta-analysis revealed that AD patients are linked with the development of HPT (Salehipour et al. 2023). Therefore, HPT could be a sequence of AD due to degeneration of hypothalamus and anterior pituitary in AD with subsequent reduction of TRH and TSH, respectively (Tan et al. 2008). Therefore, AD-induced dysmetabolism of THs aggravates AD neuropathology. A case–control study comprised 59 patients with AD or mild cognitive impairment compared to 19 healthy controls and revealed that cerebrospinal fluid (CSF) levels of THs were reduced while TSH level was increased in AD patients as compared to controls (Johansson et al. 2013). A systematic review and meta-analysis involved 32 studies revealed that serum and CSF T3 level were reduced in AD (Dolatshahi et al. 2022). These findings indicated that AD is associated with HPT.

It has been reported that AD can affect the thyroid in different ways including inhibition release of hypothalamic TRH, reduction of the sensitivity of the pituitary to the effect of TRH and depletion of TRH neurons in the hippocampus (Johansson et al. 2013). In addition, AD is correlated with high TSH levels which reduce cerebral blood flow (Chen et al. 2013; Johansson et al. 2013). However, TSH level is reduced in AD as confirmed by a case–control study (Chen et al. 2013). A postmortem study showed that TRH level was reduced in AD patients compared to controls (Chen et al. 2013). Of note, THs are transported into the central nervous system (CNS) via monocarboxylate transporter (MCT) which is downregulated in AD (Tang et al. 2019). Thus, AD adversely affects thyroid function through interruption of the physiological loop by inhibition of TRH, TSH and transport of THs into the CNS.

A cross-sectional study observed that the total T3 and T3/T4 ratios were reduced in AD patients compared to controls (Quinlan et al. 2020). Therefore, AD is linked with significant alterations of THs due to inhibition of the peripheral conversion of T4 to T3. In early AD, total T4 and TSH CSF and serum levels were reduced and increased, respectively (Johansson et al. 2013). Though, in advance AD, CSF total T3 level was highly reduced. A case–control study on 21 AD patients and 18 healthy controls confirmed that CSF levels of reverse T3 and T4 were increased as compared to controls (Sampaolo et al. 2005). This verdict suggests abnormal THs metabolism in the brain of AD patients.

**Fig. 3:**
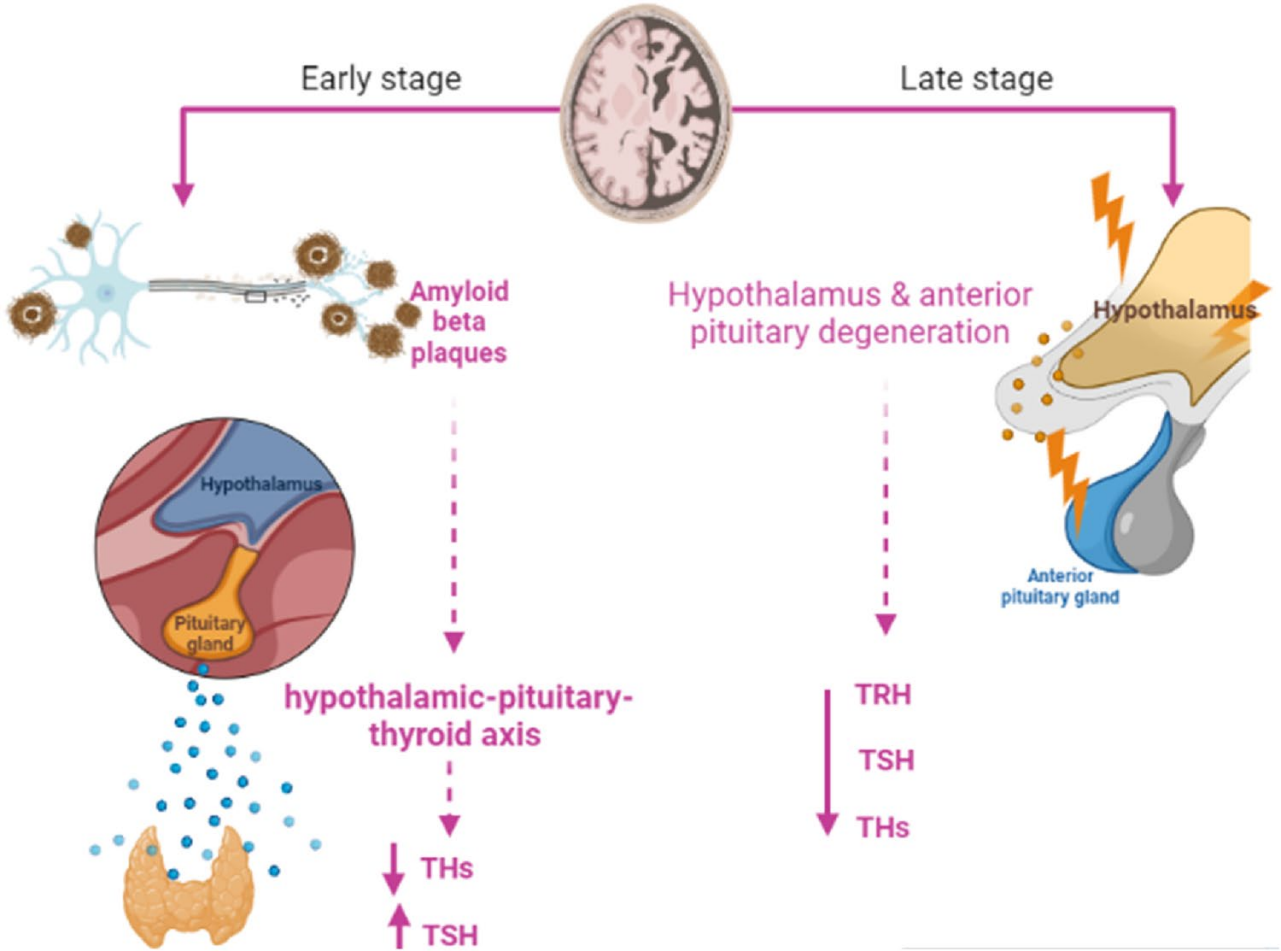
Effects of AD on thyroid function: In the early phase of AD, accumulation of A*β* induces dysregulation of the hypothalamic-pituitary-thyroid axis leading to a reduction of thyroid hormones (THs) and an increase in TSH level. In AD, both thyroid-releasing hormone (TRH) and thyroid-stimulating hormone (TSH) are reduced with subsequent reduction of THs release due to a reduction in the sensitivity of the pituitary to the effect of hypothalamic TRH. In advanced AD, degeneration of the hypothalamus and anterior pituitary reduced circulating levels of TRH and TSH with subsequent reduction of circulating THs

In the brain, the metabolism of THs is regulated by deiodinase type 2 (D2) which converts T4 to T3, and deiodinase type 3 (D3) which converts T4 and T3 to inactive metabolites, reverse T4 and reverse T3, respectively (Irachi et al. 2021). In euthyroid status, 80% of brain T3 is derived from central deiodination, and 20% is derived from peripheral deiodination (Irachi et al. 2021). In addition, reverse T3 inhibits D2 thereby reducing active T3 in the brain (Sabatino et al. 2021). D2 activity and its expression are regulated by astrocytes which are affected in AD neuropathology (MacDonald et al. 2019; Preman et al. 2021). Expression of brain D2 and D3 is highly altered in AD toward the increasing expression of D3 with downregulation of D2 (Irachi et al. 2021). Besides, the reverse T3/T4 ratio is increased in AD suggesting the development of local hypothyroidism. Of note, AD-associated pro-inflammatory cytokines could be the possible cause for the increased expression of D3 and subsequent production of reverse T3 (Lado-Abeal 2015). Reduction of brain active T3 promotes expression of the APP gene in AD patients (Accorroni et al. 2020). These observations suggest that AD triggers the development of either systemic HPT or local brain HPT (Fig. 3).

## Transthyretin in Hypothyroidism and AD

Transthyretin (TTR) which transports T4 from the circulation into the CNS through the blood choroid plexus barrier is implicated in the pathogenesis of AD. TTR is a 55kDa tetrameric protein synthesized from choroidal epithelial cells and released into the CSF. The main function of TTR is the uptake of T4 and the distribution of HPT into the CNS (Alshehri et al. 2020). TTR maintains A*β* in a soluble form and prevents aggregation of neurotoxic A*β* (Li and Buxbaum 2011). In advanced AD, CSF TTR is reduced due to atrophy of the choroid plexus (Gião et al. 2020). TTR is not necessary for the homeostasis of THs as evidenced by an experimental study (Sousa et al. 2005). Conversely, TTR is important for the homeostasis of circulating THs. In addition, TTR transports vitamin A binding protein (retinol-binding protein), and has a proteolysis effect on apolipoprotein A-I (Apo-AI), neuropeptide Y which is involved in the regulation of A*β* (Saponaro et al. 2020a). THs promote the expression of TTR (Morgado et al. 2007). In TTR-null mice, the distribution of THs is affected and characterized by low total T4 with normal free T4 and T3 (Palha et al. 2002), suggesting that deficiency of TTR induces a state of hypothyroxinemia but with euthyroid status. Remarkably, TTR regulates THs in the choroid plexus but not in the brain, as the absence of TTR does not affect the brain metabolism of THs. Therefore, there is strong controversy regarding the role of TTR in the regulation of thyroid function.

**Fig. 4:**
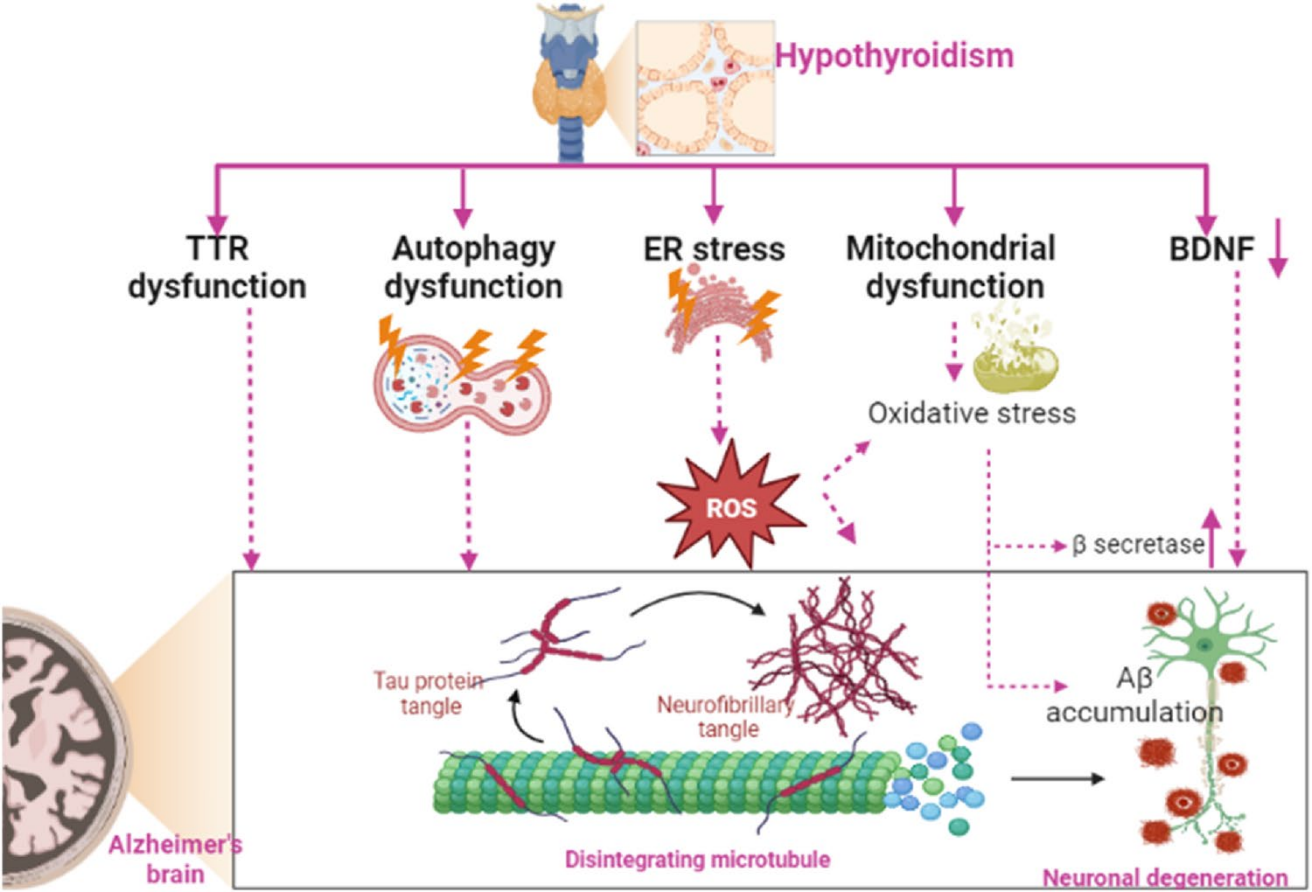
Effect of hypothyroidism (HPT) on Alzheimer’s disease. HPT-induced transthyretin (TTR) and autophagy dysfunction lead to the accumulation of amyloid beta (A*β*) that induce cognitive dysfunction and the progression of AD neuropathology by increasing neuronal degeneration. Additionally, HPT-induced ER stress and mitochondrial dysfunction with subsequent development of oxidative stress that enhances the expression of beta-secretase and generation of A*β*. HPT is associated with a decrease in BDNF levels leading to synaptic and cognitive dysfunction

It has been shown that TTR has a protective effect against the development of AD through the binding and sequestering of A*β* with a proteolytic effect (Sousa et al. 2007). Therefore, TTR CSF level was reported to be reduced with significant alterations in its expression in AD patients (Sousa et al. 2007). A case–control study on 90 AD patients and 50 healthy controls showed that TTR plasma level was reduced in AD patients as compared to controls (Velayudhan et al. 2012). Thus, TTR plasma level is regarded as a diagnostic biomarker of AD. Li and Buxbaum (2011) illustrated that TTR can abrogate A*β* deposition and AD progression. A case–control study observed that TTR plasma level was reduced in AD (*n* = 111) as compared to controls (*n* = 90) (Han et al. 2011). Notoriously, TTR is regarded as a potential biomarker for the prediction of AD (Tien et al. 2019). Noteworthy, AD risk is low in women as compared to men due to higher estrogen levels which induce the expression of a neuroprotective TTR (Quintela et al. 2009). Therefore, HPT or mutation of TTR promotes the development and progression of AD (Fig. 4).

## The Association Between AD and Hypothyroidism

### Autophagy

Autophagy is a cellular process involved in the clearance of misfolded proteins and injured organelles (Amaravadi et al. 2019). THs improve the autophagy pathway and mitochondrial biogenesis (Zhou et al. 2021). Therefore, dysregulation of the expression of THs inhibits the autophagy pathway (Vishwakarma et al. 2023). Conversely, a recent experimental study showed that the autophagy pathway is exaggerated in HPT leading to progressive neuronal injury (Mishra et al. 2021). The autophagy pathway in different thyroid diseases is dysregulated even with the same disease but with different stages (Song et al. 2023). In addition, hyperthyroidism triggers autophagy activation (Venediktova et al. 2022). Therefore, an optimal level of THs is required for autophagy function. Besides, the autophagy pathway is intricate in the pathogenesis of AD. An autophagy deficit is developed in the early stage of AD causing accumulation of A*β* (Li et al. 2017). Autophagy activators like rapamycin could be effective in AD by inducing A*β* clearance via the autophagy pathway (Cai and Yan 2013). However, an exaggerated autophagy pathway may increase the generation of A*β*, as autophagic vacuoles contain all machinery constituents for the synthesis of A*β*. APP, PS1, and secretase enzymes are found within autophagic vacuoles (Tung et al. 2012) thus activation of autophagy increases A*β* production and increases AD risk. Therefore, the autophagy pathway plays a double-edged sword in AD pathogenesis. Therefore, HPT-induced autophagy dysfunction may induce cognitive dysfunction and the progression of AD neuropathology (Fig. 4).

### Endoplasmic Reticulum (ER) Stress

HPT induces the development of hippocampal endoplasmic reticulum (ER) stress and the generation of reactive oxygen species (ROS) (Torres-Manzo et al. 2018). In addition, subclinical HPT induces dyslipidemia through the induction of oxidative stress (Zhou et al. 2016). ER stress-induced ROS promotes neurodegeneration (Doyle et al. 2011). ER stress is intricate in AD neuropathology due to the higher expression of APP and PS1 genes within ER (Hashimoto and Saido 2018). ER stress is developed in AD due to the accumulation of misfolded proteins within the ER leading to the release of unfolded protein response (UPR) as a compensatory mechanism to abrogate ER stress (Li et al. 2015). Findings from postmortem humans and animals showed that ER stress was correlated with AD neuropathology (Li et al. 2015). Therefore, HPT-induced ER stress might be a possible mechanism for the development of AD (Fig. 4).

### Brain-Derived Neurotrophic Factor

BDNF is a growth factor belonging neurotrophic factor family involved in neuronal growth and differentiation. BDNF is highly expressed in different brain regions including the amygdala, hippocampus, cerebral cortex and cerebellum (Girotra et al. 2022). Reduction of BDNF serum level with the development and progression of AD (Gao et al. 2022; Girotra et al. 2022). It has been suggested that BDNF mediates the effects of THs in the regulation of synaptic and cognitive function (Yajima et al. 2021). BDNF serum levels are reduced in rats subjected to anti-thyroid agents (Giannocco et al. 2021; Madhusudhan et al. 2022). In addition, BDNF serum levels are decreased in patients with HPT (Madhusudhan et al. 2022). Amelioration of BDNF by antidepressant agents and THs improves depressive symptoms which are commonly observed in patients with HPT (Maglione et al. 2022). Thus, the reduction of BDNF in HPT could be a causal factor for the development and progression of AD.

### Mitochondrial Dysfunction

Mitochondrial dysfunction is intricate with the development of HPT and AD (Videla and Valenzuela 2022). THs regulate the expression of mitochondrial genes and mitochondrial transcription factor A which is reduced in HPT patients as compared to healthy controls (Videla and Valenzuela 2022). T3 promotes mitochondrial genes and associated promoters including cytochrome c, therefore HPT is linked with the development of mitochondrial dysfunction due to the diminution of the bioenergetics effects of THs (Cioffi et al. 2022). Thus, using of thyromimetic agents such as sobetirome could be effective in HPT by improving mitochondrial oxygen consumption (Saponaro et al. 2020b). Likewise, the analogue of THs such as TRC150094 which is used in hepatic steatosis (Di Munno et al. 2021) could be effective in the management of HPT -related complications. Furthermore, mitochondrial dysfunction triggers AD neuropathology by enhancing tau phosphorylation and deposition of A*β* (Geib et al. 2021). As well, mitochondrial dysfunction-induced oxidative stress enhances the expression of beta-secretase, generation of A*β*, and augmentation of A*β*42/40 ratio (Francelin et al. 2021; Keller et al. 2020). In turn, A*β* triggers mitochondrial dysfunction and oxidative which further promotes A*β* accumulation in a vicious cycle (Bello-Medina et al. 2022; Busche and Hyman 2020; Rao et al. 2020; Wang et al. 2020). These verdicts indicated that mitochondrial dysfunction could be the casual relationship between HPT and AD.

Taken together, there is a potential link between HPT and AD, as HPT adversely impacts AD neuropathology and the reverse is also true. Dysregulation of TTR, oxidative stress, ER stress and autophagy dysfunction could be the possible mechanisms for this probable association (Fig. 4).

The present review had different limitations including paucity of recent and updated clinical studies, age and gender factors were not estimated separately, and effects of thyromimetic agents were not discussed briefly in relation to AD. Most previous studies evaluated the effects of hypothyroidism and hyperthyroidism on AD risk, though the present review only discussed the mechanistic role of PHT in the induction of AD, and how AD affects the thyroid function was revised definitely. Most of the involved studies were cross-sectional and even in included prospective studies the diagnostic criteria for both AD and HPT were not assessed properly in some studies. Therefore, this review cannot give the final conclusions regarding the potential nexus between AD and HPT. Preclinical and clinical studies are recommended in this regard to clarify the link between HPT and AD, and which one is the primary event.

## Future Perspectives

Early diagnose of overt and subclinical HPT by screening of old age subjects who are risk for neurodegenerative diseases might be a preventive measure against the development of AD. Likewise, screening of AD patients for risk of HPT is mandatory. Thyromimetic agents might be a novel therapeutic strategy in treating AD mainly in patients with HPT, therefore clinical trial and prospective studies are recommended in this regard. Since, TSH/TSHR axis expressed in the brain is implicated in AD, using of TSHR modulators may open a new therapeutic era in the management of AD. Further, molecular studies are warranted to study the subcellular effects of THs in AD and other neurodegenerative diseases. Moreover, sophisticated measurements of brain THs expression in patients with PHT are needed to detect localized brain HPT regardless of primary HPT.

## Conclusions

HPT can affect AD neuropathology through various mechanistic pathways including oxidative stress, mitochondrial dysfunction, and inhibition of BDNF. HPT excites the development and progression of AD by inducing the production of A*β* and tau protein phosphorylation with subsequent dysfunction of synaptic plasticity and memory function. The metabolism of THs is dysregulated by AD due to the accumulation of A*β* leading to local brain HPT. Taken together, there is a potential nexus between HPT and AD, as HPT adversely impacts AD neuropathology and the reverse is also true. Preclinical and clinical studies are prerequisites in this regard.

## Data Availability

Not applicable.
